# Necessary Knowledge and Skills for Dietitians in Saudi Arabia: A Qualitative Study

**DOI:** 10.21315/mjms2019.26.3.9

**Published:** 2019-06-28

**Authors:** Khalid Aldubayan, Ghadeer Aljuraiban, Dara Aldisi

**Affiliations:** Department of Community Health Sciences, College of Applied Medical Sciences, King Saud University, P.O. Box 10219, Riyadh, Kingdom of Saudi Arabia

**Keywords:** knowledge and skills, dietitians, clinical nutrition, focus group, Saudi Arabia

## Abstract

**Background:**

Dietitians play a major role in health promotion and chronic diseases prevention. Graduates from clinical nutrition and dietetics major should be equipped with the necessary knowledge and skills for their role to be more effective. The purpose of this study is to investigate the knowledge and skills needed by current and future graduates in clinical nutrition and dietetics.

**Methods:**

In this qualitative study, structured interviews by focus groups were conducted. Dietitians from different governmental and private sectors were invited to participate in the study. Focus groups were stratified based on the participants’ gender and years of experience to promote self-disclosure. Abridged transcript of relevant and useful points was performed. The transcripts were coded and cross-validated by two researchers.

**Results:**

A total of four focus groups were conducted. Two focus groups comprise 9 male participants and the other two comprise 10 females. The age of participants ranged 25–40 years old. Participants were employees in Riyadh city with experience that ranged 3–10 years old. Nine themes of the necessary knowledge and skills were identified.

**Conclusion:**

It is recommended for the Saudi government to create standards specialised for clinical nutrition and dietetics undergraduate and graduate programmes.

## Introduction

The prevalence of non-communicable diseases has been increasing globally. Unhealthy dietary and lifestyle habits play an important role in the increase of diet-related diseases, accounting for 63% of global deaths (1). In 2010, non-communicable diseases such as cardiovascular diseases and diabetes became the leading cause of death in Saudi Arabia, and obesity was the leading risk factor for those diseases (2). Prevention of chronic diseases by means of diet and lifestyle changes requires great emphasis on awareness. Evidence shows that nutrition-focused educational services in both clinical and community settings have positive impact on patients’ outcomes and community’s health (3–8). In Saudi Arabia, a cross-sectional study showed positive effect of nutritional counseling done by dietitians on glycemic control in diabetic patients (9). In accordance to the standards set by the Academy of Nutrition and Dietetics, the role of registered dietitians in nutrition-based preventive care is crucial in the health care system and can impact both policy and healthcare initiatives (7). On a larger scale, their role can also aid in improving dietary behaviour and lifestyle, which could have an effect on reducing the economic burden of diet related diseases (7).

Trends in healthcare demand that graduates in clinical nutrition and dietetics be equipped with the necessary knowledge and skills to adapt to the evolving healthcare environment. The increasing prevalence in chronic diseases, especially among the aging population, has stimulated a demand for nutrition discipline and created specific niches for specialised competent professionals (10, 11). Dietitians play a role in patient care in hospitals and outpatient centres (12, 13). This is in addition to contributing in health promotion by taking part in government sector and private practice (12, 13). Aligning the clinical nutrition curriculum in universities with the needs of stakeholders may help fulfill the demands of the workplace and the expectations of graduates in clinical nutrition. Universities play a vital role in the development of work-ready graduates that allows for a smooth transition from student to health practitioner, especially for emerging health professions (14).

Here, we identify the knowledge and skills needed by current and future graduates in clinical nutrition and dietetics in a qualitative study. The information was extracted from interviews conducted using focus groups composed of currently employed clinical nutritionists. The focus groups provided an opportunity to gather information from qualified professionals in the field of clinical nutrition/ dietetics who gave feedback on the challenges they encountered as either trainers, supervisors or employers of newly graduated clinical nutritionists.

## Methodology

This is a qualitative study that used the focus group technique to gather information that provide insights of employees specialised in nutrition. This study is regarding to the knowledge and skills needed for dietitians in Saudi Arabia. Purposive sampling was used to select participants specialised in nutrition to invite them until reaching saturation. In order to promote self-disclosure, we stratified participants based on their gender and years of experience in the field.

Participants were recruited by sending official invitation letters to them through their institution. They were selected to represent different governmental and private sectors such as hospitals, clinics and organisations in Riyadh city to reach different perceptions.

The focus groups were conducted in a meeting room at the Community Health Sciences Department at the College of Applied Medical Sciences, King Saud University. Three researchers with nutrition qualifications moderated the focus groups according to participants’ gender. The moderators had some previous experience in conducting focus groups. The researchers followed Krueger and Casey’s five categories in designing questions that aid in organising the flow of a focus group (15). These categories are: Opening, Introductory, Transition, Key and Ending with more depth in Key category. The final questions were reviewed by all researchers. The focus group discussion guide consisted of 10 questions (1 Opening, 1 Introductory, 1 Transition, 5 Keys, 2 Endings). The focus groups were audio recorded and its duration ranged from 60–90 min. The audio recordings were subsequently used to transcribe the discussions for analysis. The institutional review board (IRB) at King Saud University granted ethical approval for this study. Signed consent forms were received from participants before commencing with the focus groups.

As the moderators have nutritional backgrounds, abridged transcript of relevant and useful points was performed. Field notes were used as well for noting non-verbal communications. The transcripts were translated from the Arabic language to the English language. The information in the transcripts and notes was coded by two researchers and then the analyses were cross-validated.

## Results

Four focus groups that consisted of 19 participants (9 males and 10 females) aged 25–40 years old were conducted. All participants were specialised in nutrition and their experience ranged 3–10 years old. All participants work in Riyadh city. Nine themes of the data were identified: food and nutrition policy, food and drug interactions, nutrition care process, research methods and statistics, nutrition for pediatric, enteral and parenteral feeding, communication and counseling, evidence-based practices, and sport nutrition. Each theme was supported with the participants’ comments, quotes, and recommendations (Table 1).

Male participants focused more on the requirements of knowledge and skills regarding regulations and legislations in food and nutrition, whereas female participants were more concerned about dealing with pediatric patients. Participants frequently mentioned the need for research skills and how these skills can be applied as well as integrating research into evidence-based practices. In addition, the need for professional communication skills was frequently highlighted by participants. This is in addition to the importance of having clinical practices in the nutrition care process, and enteral and parenteral feeding were noted. It was also mentioned that having a background in food and drug interactions, sport nutrition, and human psychology and behaviours were essential for dietitians.

## Discussion

The main discussion topic in our focus group of 19 dietitians’ concerned inadequacies of the clinical nutrition programme to provide dietetic graduates with the necessary skills that would allow them to affect hospital and public policy. Courses on food and nutrition policy were deemed important to include in the course curriculum of dieticians at the universities. This is in line with the ‘standards of practice and professional performance for registered dietitians of the American Dietetic Association (ADA) (16). Their report outlines the responsibilities of dietitians to work with public policy in ensuring high-quality food and nutrition services in healthcare. Dietitians need the knowledge and skills that would allow them to interact with policymakers and public health leaders on nutrition-related health issues.

Focus group participants generally agreed that the skills which the graduates lacked were oral and written communication skills. Previous recommendations and reports in the dietetics field have emphasised communication and counseling as important skills for improving patient care (17, 18). The diversity of the Saudi population makes an understanding of the various needs of the population essential to ensure patients’ compliance with the recommendations of the dietitians, especially compliance by critically ill patients (19). Previous investigations have suggested that incorporating video simulations and computer-assisted instruction into the dietetics curriculum improves the communication skills of the students (18, 20). The Litchfield et al. pilot study evaluated a model of interactive communication technology among dietetic graduates and reported that non-traditional methods of communication improved the competency of graduates and heightened their learning skills (21).

Another focus group concern was the low level of knowledge often encountered among graduates in the area of advanced nutrition assessment tools, like biochemical markers, and appropriate, corresponding interventions. Group participants also observed that graduates seldom implement the principles of the NCP during patient care and suggested that the NCP should be implemented throughout the dietetics bachelor’s degree programme. Focus group participants observed that many graduates had a low level of knowledge in food and drug interactions, had a low level of practice in enteral and parenteral feeding, especially of intensive care patients, and had sub-optimal skills in pediatric nutrition. Health professionals such as dietitians should be aware of possible food and drug interactions, as some drugs can have an influence on nutrients’ absorption and metabolism, and, on the other hand, some foods or supplements can interact with drugs efficiency (22). Taylor and colleagues critically reviewed the role of the dietitian in the intensive care unit and have defined dietitians as clinicians, educators, and researchers who should be well competent to practice in a multidisciplinary team approach (23). This approach was found to reduce costs and improve patient care. For this to succeed, authors of the review emphasised that the dietitian must be equipped with advanced training in critical care nutrition. They also encouraged dietitians to think outside the box and be innovative in dealing with the side effects of enteral and parenteral feeding, and when dealing with pediatric patients, a practice that can only be mastered with high level and efficient training (23). A recent evaluation of implementation of the NCP into dietetics curricula showed that it significantly improved the competencies of graduates and their knowledge domains (24).

Focus group participants commented that dietetic graduates often lack skills and practices in scientific research, especially statistical knowledge. In their observations, dietetic graduates have poor skills in the critical thinking necessary for reading scientific articles and translating them correctly into practice. A qualitative study in 2018 by Hinrichs examined the perceptions of dietetic intern on evidence-based medicine; although interns considered evidence-based medicine important for their careers, they found integrating research with practice to be challenging (25). The Academy of Nutrition and Dietetics Career Development Guide emphasises the importance of critical thinking skills to ensure appropriate interpretation of data, a skill not possible to obtain unless interns are well-equipped with advanced research skills (26). The ADA has previously stressed the importance of integrating research skills into the coursework of dietetic interns and suggested models that mandate research based on ADA knowledge and performance requirements (27). These improvements in dietetic graduate training would help graduates better communicate ways of improving general health of their patients; public policy would gain through future decreases in general healthcare costs.

The participants of the focus group emphasised the need to equip dietitians with the knowledge and skills of sport nutrition. Globally, nutrition has been increasingly recognised as an important component in reaching optimal performance and fast recovery (28–30). In USA, a cross-sectional study (579 participants) showed that registered dietitians were the primary nutrition resources for coaches and athletes (31). According to the Saudi Quality of Life Program, the objective of the 2030 Vision for professional sports dimension is to reach regional and global excellence in selected professional sports (32). This trend increases the demand of dietitians graduated with sufficient knowledge and skills in sport nutrition.

To the best of the authors’ knowledge, this is the first study that identifies the necessary knowledge and skills for dietitians by using a qualitative method. However, the generalisability of the study’s results is limited. Further quantitative studies are required.

## Conclusion

Identifying the knowledge and skills needed and the trends in the workplace could lead to curriculum revisions by the universities aiming to train professionals with stronger competencies to meet the changing healthcare needs of the population. Our studies showed the needs of establishing standards for nutrition and dietetics practices by the Saudi government. In Saudi Arabia, universities have been seeking academic accreditation to ensure competent graduates. It is recommended that the National Commission for Academic Accreditation and Assessment (NCAAA) cooperate with the Saudi Commission for Health Specialties (SCHS) to create such specialised standards.

## Figures and Tables

**Table 1:** Themes regarding necessary knowledge and skills for dietitians in Saudi Arabia

Theme 1: Food and Nutrition Policy		
Comments	Quotes	Recommendations
Participants, mainly males, mentioned the importance of having background information and skills in the policymaking process in the field of food and nutrition.	*‘Bachelor graduates lack skills in writing legislation’ MFG1*^a^ *‘Understanding both the national and international laws and regulations for food and nutrition are important, such as the front-of-pack food labeling and nutrition and health claims’ MFG^1^a*	It is important to include in nutrition academic programmes with sufficient components regarding food and nutrition policy.
**Theme 2: Food and Drug Interactions**		
**Comments**	**Quotes**	**Recommendations**
Participants mentioned the importance of understanding the possible interactions between nutrients and drugs.	*‘Food and drugs interactions is an essential subject’ MFG1*^a^ *‘Some drugs interact with nutrients that affect its absorption and vice-versa, therefore, it is important to understand that’ FFG2*^a^	Nutrition academic programmes should include basic information regarding food and drug interactions.
**Theme 3: Nutrition Care Process**		
**Comments**	**Quotes**	**Recommendations**
Participants mentioned the importance of practicing the use of different nutrition assessment tools and designing appropriate interventions.	*‘Need to have a better understanding of biomarkers and their lab tests’ FFG1*^a^ *‘We need more training on the tools that are used for physical examination and anthropometric measurements’ MFG1*^a^ *‘Follow nutrition care process model’ FFG2*^a^ *‘How to design a meal plan more appropriate to patients’ culture’ MFG2*^a^	Including information regarding principles of Nutrition Care Process (NCP) is essential throughout a nutrition bachelor programme.
**Theme 4: Research Methods & Statistics**		
**Comments**	**Quotes**	**Recommendations**
Participants emphasized on the importance of equipping students with research skills. Participants mentioned frequently that dietitians lack statistical knowledge and skills.	*‘It is important to know how to design research studies’ FFG1*^a^ *‘To better the understanding of studies’ results, we need to have background in statistics’ MFG1*^a^ *‘Programmes should not be focused only on the clinical part, as graduates needs to have knowledge and skills in research’ MFG1*^a^ *‘It is essential to understand different types of data and their appropriate statistical tests’ FFG2*^a^ *‘We need to understand research methodology and how to interpret results properly’ MFG2*^a^ *‘We have access to data in our workplace, but we do not have the skills to analyse them and interpret them the properly’ MFG2*^a^	Nutrition academic programmes should provide sufficient components on research methodology and statistics to prepare dietitians in data analysis and interpretation.
**Theme 5: Nutrition for Pediatric**		
**Comments**	**Quotes**	**Recommendations**
Participants mainly females mentioned the importance of having practical background information on nutrition for pediatrics.	*‘We need more training in pediatrics’ FFG1*^a^ *‘We struggled in dealing with pediatric patients, and some interns were not confident during pediatric ward rounds’ FFG2* a	It is recommended for nutrition academic programmes to increase the focus on pediatrics throughout their programme.
**Theme 6: Enteral and Parenteral Feeding**		
**Comments**	**Quotes**	**Recommendations**
Participants highlighted that working at an intensive care unit requires having a practical background in enteral and parenteral feeding.	*‘It is important for practitioners to know appropriate tube feeding for patients in the critical care unit’ MFG1*^a^ *‘One of the standards for accreditation of healthcare institutions in Saudi Arabia focuses on enteral and parenteral feeding in ICU’ FFG2*^a^	Nutrition academic programmes should provide sufficient components regarding enteral and parenteral feeding.
**Theme 7: Communication and Counseling**		
**Comments**	**Quotes**	**Recommendations**
Participants emphasized that the professional practice requires a high level of oral and written communication skills. Participants mentioned the importance of group work and counseling skills.	*‘There is a gap on how to deal with patients properly, especially those who are difficult, and the need have effective communication with them’ FFG1*^a^ *‘We need better understanding of human psychology and behaviours’ FFG1*^a^ *‘Graduate writing skills need improvement in terms of structure, depth of the content and proper citations’ MFG1*^a^ *‘Some graduates lack team work skills’ FFG2*^a^ *‘Our role in some hospitals are unclear, which may cause miscommunication between us and other health practitioners’ MFG2*^a^	Nutrition academic programmes should prepare their students with different kinds of communication skills throughout the programme. Nutrition and dietetics practices such as the scope of practice, code of ethics for the profession, and interprofessional relationships should be governed. It is suggested that including some components in human psychology and behaviour theories are required for nutrition academic programmes.
**Theme 8: Evidence-based Practices**		
**Comments**	**Quotes**	**Recommendations**
Participants highlighted the challenge that dietitians may face in reading scientific articles and translating them appropriately into practice.	*‘We need to read more scientific articles and come up with suitable recommendations’ FFG1*^a^ *‘It is important to practice the use of critical appraisal tools’ MFG1*^a^ *We need to know more on how to evaluate the strength of evidence of research and the differences between studies’ MFG2*^a^	Nutrition academic programmes should provide sufficient components about integrating research principles into evidence-based practices.
**Theme 9: Sport Nutrition**		
**Comments**	**Quotes**	**Recommendations**
Participants mentioned the importance of preparing students with sports nutrition background information as it becomes a new trend in Saudi Arabia.	*‘We need to focus more on sports nutrition’ FFG2* a *‘Sports nutrition is the new trend in Saudi Arabia and we need to be prepared’ MFG2*^a^	Increasing the focus on sports nutrition in nutrition academic programmes is recommended.

Note:

a FFG = Female Focus Group, MFG = Male Focus Group

## References

[1] World Health Organization (2013). 2013–2020 Globle action plan for the prevention and control of noncommunicable diseases. [Internet].

[2] Memish ZA, Jaber S, Mokdad AH, AlMazroa MA, Murray CJ, Al Rabeeah AA (2014). Peer reviewed: burden of disease, injuries, and risk factors in the Kingdom of Saudi Arabia, 1990–2010. Prev Chronic Dis.

[3] Baute V, Sampath-Kumar R, Nelson S, Basil B (2018). Nutrition education for the healthcare provider improves patient outcomes. Global Advances in Health and Medicine.

[4] Holmes AL, Sanderson B, Maisiak R, Brown A, Bittner V (2005). Dietitian services are associated with improved patient outcomes and the MEDFICTS dietary assessment questionnaire is a suitable outcome measure in cardiac rehabilitation. J Am Diet Assoc.

[5] Kahan S, Manson JE (2017). Nutrition counseling in clinical practice: how clinicians can do better. JAMA.

[6] Schiller MR, Miller M, Moore C, Davis E, Dunn A, Mulligan K (1998). Patients report positive nutrition counseling outcomes. J Am Diet Assoc.

[7] Slawson D, Fitzgerald N, Morgan K (2013). Position of the Academy of Nutrition and Dietetics: the role of nutrition in health promotion and chronic disease prevention. J Acad Nutr Diet.

[8] Tappenden KA, Quatrara B, Parkhurst ML, Malone AM, Fanjiang G, Ziegler TR (2013). Critical role of nutrition in improving quality of care: an interdisciplinary call to action to address adult hospital malnutrition. J Parenter Enteral Nutr.

[9] Alshareef SM, Alkhathlan MA, Alwabel AA, Al-Bawardi AA, Alqarni AH, Almuryidi AS (2018). How does the utilization of diabetes dietitian and educator service in Saudi Arabia affect glycemic outcomes?. J Family Community Med.

[10] Gaba A, Shrivastava A, Amadi C, Joshi A (2016). The nutrition and dietetics workforce needs skills and expertise in the New York metropolitan area. Glob J Health Sci.

[11] Kris-Etherton PM, Akabas SR, Douglas P, Kohlmeier M, Laur C, Lenders CM (2015). Nutrition Competencies in Health Professionals’ Education and Training: A New Paradigm. Advances in Nutrition.

[12] Olstad DL, Raine KD, McCargar LJ (2013). The role of registered dietitians: in health promotion. Can J Diet Pract Res.

[13] Rogers D (2018). Compensation and benefits survey 2017. J Acad Nutr Diet.

[14] Almoayad F, Ledger A (2016). Entering a new profession: Patient educator interns’ struggles for recognition. J Health Spec.

[15] Krueger RA, Casey MA (2002). Designing and conducting focus group interviews [Internet].

[16] Robinson GE, Cryst S (2018). Academy of nutrition and dietetics: revised 2018 standards of practice and standards of professional performance for registered dietitian nutritionists (competent, proficient, and expert) in post-acute and long-term care nutrition. J Acad Nutr Diet.

[17] Lawrence J (2003). Communication and education skills for dietetics professionals. J Hum Nutr Diet.

[18] Pignone MP, Ammerman A, Fernandez L, Orleans CT, Pender N, Woolf S (2003). Counseling to promote a healthy diet in adults: a summary of the evidence for the US Preventive Services Task Force. Am J Prev Med.

[19] Curtis JR, Back AL, Ford DW, Downey L, Shannon SE, Doorenbos AZ (2013). Effect of communication skills training for residents and nurse practitioners on quality of communication with patients with serious illness: a randomized trial. JAMA.

[20] Raidl MA, Wood OB, Lehman JD, Evers WD (1995). Computer-assisted instruction improves clinical reasoning skills of dietetics students. J Am Diet Assoc.

[21] Litchfield RE, Oakland MJ, Anderson JA (2000). Improving dietetics education with interactive communication technology. J Am Diet Assoc.

[22] Mason P (2010). Important drug–nutrient interactions. Proc Nutr Soc.

[23] Taylor B, Renfro A, Mehringer L (2005). The role of the dietitian in the intensive care unit. Curr Opin Clin Nutr Metab Care.

[24] Karupaiah T, Reinhard T, Krishnasamy S, Tan SP, Se CH (2016). Incorporating the nutrition care process model into dietetics internship evaluation: a Malaysian university experience. Nutr Diet.

[25] Hinrichs RJ (2018). Dietetic interns’ perceptions and use of evidence-based practice: an exploratory study. J Med Libr Assoc: JMLA.

[26] Charney P, Peterson SJ (2013). Practice paper of the academy of nutrition and dietetics abstract: Critical thinking skills in nutrition assessment and diagnosis. J Acad Nutr Diet.

[27] Hynak-Hankinson MT, Martin S, Wirth J (1997). Research competencies in the dietetics curricula. J Am Diet Assoc.

[28] Rodriguez NR, Di NM, Langley S (2009). American College of Sports Medicine position stand. Nutrition and athletic performance. Medicine and Science in Sports and Exercise.

[29] Burke LM, Meyer NL, Pearce J (2013). National nutritional programs for the 2012 London Olympic Games: a systematic approach by three different countries. Limits of Human Endurance.

[30] Thomas DT, Erdman KA, Burke LM (2016). Position of the academy of nutrition and dietetics, dietitians of Canada, and the American College of Sports Medicine: nutrition and athletic performance. J Acad Nutr Diet.

[31] Torres-McGehee TM, Pritchett KL, Zippel D, Minton DM, Cellamare A, Sibilia M (2012). Sports nutrition knowledge among collegiate athletes, coaches, athletic trainers, and strength and conditioning specialists. J Athl Train.

[32] Vision 2030 (2018). Quality of life program 2020. [Internet].

